# Short-term effect of polyethylene glycol loxenatide on weight loss in overweight or obese patients with type 2 diabetes: An open-label, parallel-arm, randomized, metformin-controlled trial

**DOI:** 10.3389/fendo.2023.1106868

**Published:** 2023-01-26

**Authors:** Hongyu Cai, Qianqian Chen, Yale Duan, Yue Zhao, Xiujuan Zhang

**Affiliations:** ^1^ Department of Endocrinology, Shandong Provincial Hospital Affiliated to Shandong First Medical University, Jinan, Shandong, China; ^2^ Department of Medical Affairs, Jiangsu Hansoh Pharmaceutical Group Co., Ltd., Shanghai, China

**Keywords:** polyethylene glycol loxenatide, weight loss, overweight, obesity, type 2 diabetes, glucagon-like peptide 1 receptor agonist, metformin

## Abstract

**Objective:**

Polyethylene glycol loxenatide (PEG-Loxe) is a novel, once-weekly glucagon-like peptide 1 receptor agonist that is approved in doses of 0.1 mg and 0.2 mg for the treatment of type 2 diabetes mellitus (T2DM). However, no clinical trials have been designed to determine the effect of 0.3 mg PEG-Loxe on weight loss in overweight or obese patients with T2DM. This trial aimed to evaluate the short-term effect of 0.3 mg PEG-Loxe, injected subcutaneously once weekly, for weight management in overweight or obese patients with T2DM.

**Methods:**

This 16-week, open-label, parallel-arm, randomized, metformin-controlled trial was conducted at Shandong Provincial Hospital in Shandong, China. Patients with T2DM, who were overweight or obese (body mass index ≥ 25.0 kg/m^2^) and had been treated with lifestyle interventions or a combination with oral antidiabetic drug monotherapy were randomized (2:1) to receive 0.3 mg PEG-Loxe or 1500 mg metformin. The primary endpoint was a change in body weight from baseline to week 16.

**Results:**

Overall, 156 patients were randomized and exposed to treatment. Weight loss was 7.52 kg (8.37%) with PEG-Loxe and 2.96 kg (3.00%) with metformin, with a between-group difference of 4.55 kg (95% CI, 3.43 to 5.67) (*P* < 0.001). A significantly higher proportion of patients lost ≥5% (61.5% vs. 25.0%) or 10% (26.9% vs. 5.8%) body weight in the PEG-Loxe group than in the metformin group (*P* < 0.01). Additionally, PEG-Loxe resulted in marked improvements in several cardiovascular risk factors compared to metformin, including body mass index, waist circumference, visceral fat area, blood pressure, and lipid profile. PEG-Loxe and metformin displayed almost equal potency for glycemic control. The incidence of adverse events was 46.2% (48/104) and 44.2% (23/52) in the PEG-Loxe and metformin groups, respectively.

**Conclusion:**

In overweight or obese patients with T2DM, a once-weekly subcutaneous administration of PEG-Loxe for 16 weeks, in addition to lifestyle interventions or oral antidiabetic drug therapy, resulted in significantly greater weight loss compared to metformin. Additional trials are necessary to establish whether these effects can be maintained in the long term.

**Clinical trial registration:**

www.chictr.org.cn, identifier ChiCTR2200057800.

## 1 Introduction

Type 2 diabetes mellitus (T2DM) is commonly associated with being overweight or obese. In China, more than half of patients with T2DM are overweight or obese (1). These conditions are associated with an increased risk of poor glycemic control, hypertension, dyslipidemia, and cardiovascular disease in patients with T2DM (2, 3).

Weight management is a vital aspect of treatment for patients with T2DM. However, weight gain is a side effect of some antidiabetic drugs, including thiazolidinedione, sulfonylurea, glinide, and insulin (4–6). Therefore, for overweight or obese patients with T2DM, antidiabetic drugs causing weight loss would be the preferred strategy for managing diabetes. Strong evidence shows that metformin, sodium-glucose cotransporter 2 inhibitors (SGLT2i), and glucagon-like peptide 1 receptor agonists (GLP-1RAs) can induce weight loss while improving glycemic control (7, 8).

GLP-1RAs are a relatively new class of drugs used to treat T2DM in the general population. The primary function of GLP-1RA is to improve glucose metabolism by increasing pancreatic β-cell insulin secretion and reducing α-cell glucagon secretion. Another well-known effect of GLP-1RA is weight loss through appetite suppression and reduced food intake (9). In addition to their roles in glycemic control and weight loss, several GLP-1RAs were shown to reduce the risk of major adverse cardiovascular events in patients with T2DM (10–13).

Polyethylene glycol loxenatide (PEG-Loxe) is a novel GLP-1RA derived from exendin-4, with 53% homology to human GLP-1 and an anti-PEG-Loxe antibody positive rate of < 2% (14–16). PEG-Loxe is approved in once-weekly doses of 0.1 mg and 0.2 mg for the treatment of T2DM and has been proven to be efficacious and well tolerated (15, 16). As a secondary endpoint, weight loss of <1 kg was observed in non-obese patients with T2DM administered PEG-Loxe doses in phase 3 trials (15, 16). In a phase 1 trial, administration of 0.3 mg PEG-Loxe resulted in a weight change of -1.9 kg in non-obese patients with T2DM (17). However, no clinical trials have been designed to determine the effect of this dose on weight loss in overweight or obese patients with T2DM. Therefore, this trial aimed to evaluate the short-term effect of PEG-Loxe dose of 0.3 mg, injected subcutaneously, once weekly for weight management in overweight or obese patients with T2DM.

## 2 Materials and methods

### 2.1 Trial design and participants

This 16-week, open-label, parallel-arm, randomized, metformin-controlled trial was conducted between March 2022 and October 2022 at the Department of Endocrinology, Shandong Provincial Hospital, Shandong, China. The Ethics Committee of Shandong Provincial Hospital approved the trial protocol (No. 2022-046/February 2022), which complied with the Declaration of Helsinki. Written informed consent was obtained from all the participants. The trial was registered in the Chinese Clinical Trial Registry (ChiCTR2200057800).

Key inclusion criteria included T2DM diagnosis (according to the 1999 World Health Organization criteria) (18), 18–65 years of age, body mass index (BMI) of ≥25.0 kg/m2, hemoglobin A1c (HbA1c) of 7.0–10.0%, and treated with lifestyle interventions or in combination with a stable dose of one oral hypoglycemic drug (thiazolidinedione, sulfonylurea, glinide, or a-glucosidase inhibitor) for at least 3 months. Key exclusion criteria included type 1 diabetes, gastrointestinal disorders associated with long-term nausea and vomiting, a history of acute or chronic pancreatitis, or had been treated with any GLP-1 RA or dipeptidyl peptidase-4 inhibitor within the last 3 months. Detailed inclusion and exclusion criteria are presented in **Supplementary Figure S1**.

### 2.2 Randomization and masking

Eligible patients were randomly assigned using an Interactive Web Response System in a 2:1 ratio to receive 0.3 mg PEG-Loxe (Hansoh Pharma) or 1500 mg metformin (Merck). Randomization was stratified according to the following two variables:

- BMI: 25.0–29.9 kg/m^2^ (overweight) or ≥30.0 kg/m^2^ (obese); and
- Pre-trial treatment: lifestyle interventions or OAD therapy.

Moreover, the clinical trial statistician was blinded to the two groups during data analysis.

### 2.3 Procedures

PEG-Loxe or metformin was added to the current treatment regimen of each patient: OAD therapy or lifestyle interventions. PEG-Loxe was injected subcutaneously once weekly. This treatment followed a fixed-dose-escalation regimen: an initial dose of 0.1 mg for 4 weeks, followed by 0.2 mg for 4 weeks, then a maintenance dose of 0.3 mg for 8 weeks. Similarly, a metformin maintenance dose of 1500 mg was administered in 500 mg weekly increments from 500 mg to 1500 mg.

In cases where the baseline HbA1c level was **<**7.5%, patients taking sulfonylurea or glinide were asked to decrease their dose to minimize the risk of hypoglycemia (10). Dose adjustment was performed according to the methods of Elhadd et al. and Kendall et al. (19, 20).

Patient visits were at 4, 8, and 16 weeks, and they included physical examination and data collection. The following information was obtained: demographic data, medical history, vital signs, visceral fat area (VFA), and laboratory test results. Laboratory tests included those for HbA1c, fasting plasma glucose (FPG), C-peptide, lipid profile, and liver function. Additionally, any adverse events (AEs) were recorded. For weight measurement, patients were instructed to remain in the fasting condition, wear light clothing, and take off their shoes. Systolic blood pressure (SBP), diastolic blood pressure (DBP), and pulse rate were measured using a blood pressure monitor (HEM-7312; OMRON, Kyoto, Japan). VFA was assessed using bioelectric impedance analysis (InBody 720, Seoul, South Korea). Fasting blood samples were collected in the morning and analyzed at the Clinical Laboratory of Shandong Provincial Hospital. β-cell function (HOMA-B) and insulin sensitivity (HOMA-S) were estimated using FPG and fasting C-peptide in an updated Homeostasis Model Assessment (HOMA2) obtained from the University of Oxford database (https://www.dtu.ox.ac.uk/homacalculator/). Hypoglycemia was classified as level 1, 2, or 3 based on the definitions of the American Diabetes Association guidelines (16) (Supplement).

### 2.4 Endpoints

Endpoints were collected at week 16, and the primary endpoint was a change in body weight. The secondary endpoints included the proportion of patients with ≥5% and ≥10% weight loss percentages; and changes in BMI, waist circumference (WC), VFA, HbA1c, FPG, C-peptide, HOMA-B, HOMA-S, total cholesterol (TC), triglycerides (TG), low-density lipoprotein cholesterol (LDL-C), high-density lipoprotein cholesterol (HDL-C), SBP, and DBP. The safety endpoints included AEs, serious AEs (SAEs), hypoglycemic events, levels of alanine aminotransferase (ALT) and aspartate aminotransferase (AST), and pulse rate.

### 2.5 Statistical analysis

The planned sample size of 150 patients was randomized 2:1 to receive either PEG-Loxe or metformin. This sample size was expected to provide a power of 80% to detect a difference of ≥2 kg weight loss between the PEG-Loxe and metformin groups, with a standard deviation (SD) of 3.66 (17, 21), α level of 0.05, and 20% withdrawal rate.

The normal distribution of variables was evaluated using the Kolmogorov-Smirnov test and by assessment of residual distribution. Baseline variables were analyzed using independent t test, Mann-Whitney U test, and χ² test.

The efficacy analyses were evaluated using the full analysis set, defined as patients exposed to ≥1 treatment dose and had a baseline assessment. Safety data were assessed using the safety analysis set, defined as patients exposed to ≥1 treatment dose. The primary endpoint (change in body weight) was analyzed using a mixed model for repeated measurements (MMRM), which included group, time, and the corresponding interactions as fixed effects, and baseline weight and sex as covariates. MMRM was used to analyze BMI, WC, VFA, HbA1c, FPG, C-peptide, HOMA-B, HOMA-S, TC, TG, LDL-C, HDL-C, SBP, DBP, ALT, AST, and pulse rate. Categorical variables were evaluated using the χ² test or Fisher exact test. Missing data were imputed using a multiple linear imputation analysis according to the rules of Rubin (22). Sensitivity analyses were performed on the per-protocol set, defined as patients who completed the trial without major protocol violations.

Results are shown as adjusted mean and 95% CI, if not indicated otherwise. A *P* value < 0.05 (two-tailed) was considered statistically significant. All statistical analyses were performed using Statistical Analysis System (SAS) version 9.4 (RRID: SCR_008567).

## 3 Results

**Figure 1:**
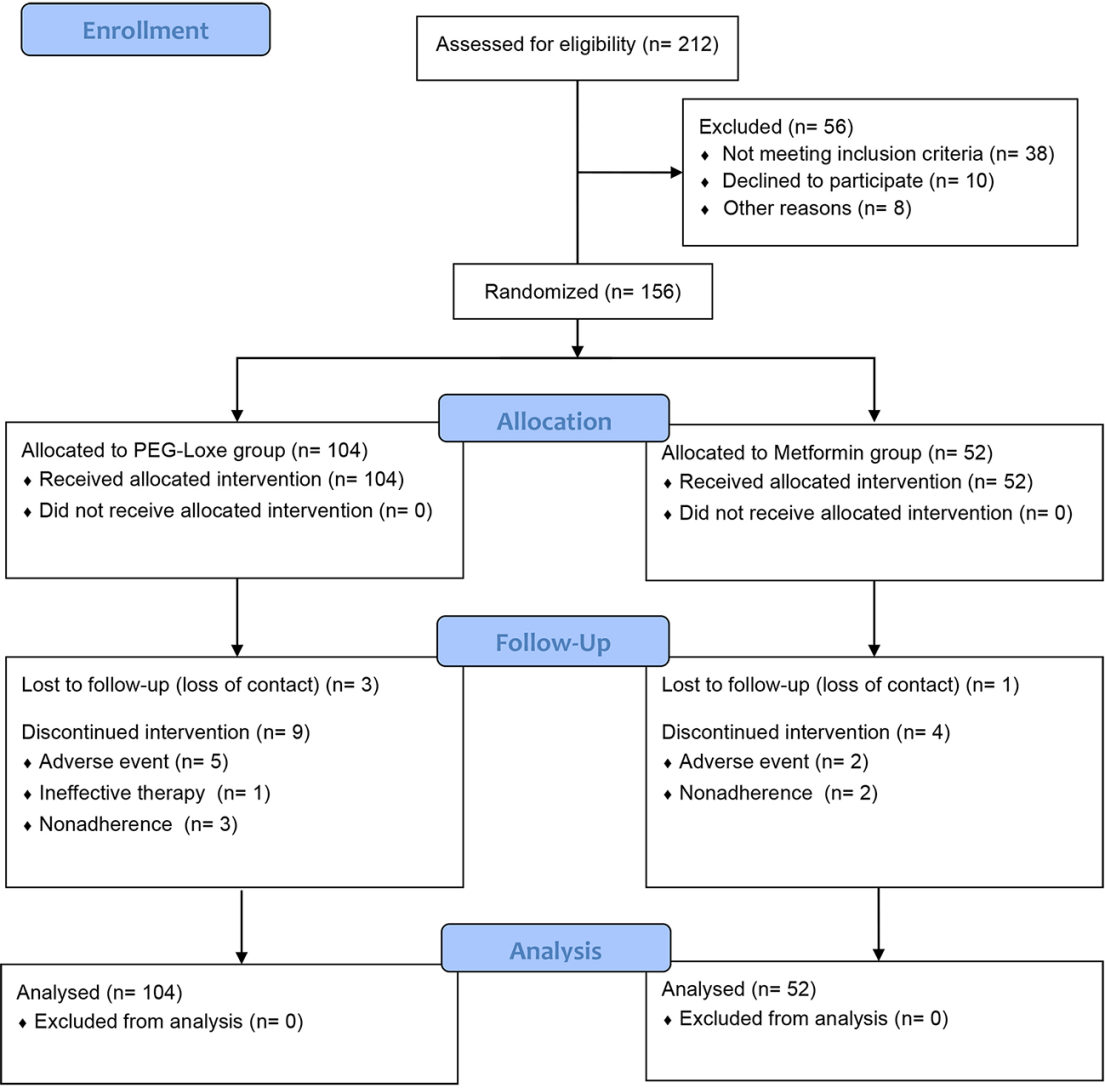
Consort flow diagram.

Between March 2022 and June 2022, 212 patients were screened, of which 156 were enrolled and randomized to receive PEG-Loxe (n = 104) or metformin (n = 52). Twelve (11.5%) patients in the PEG-Loxe group and five (9.6%) patients in the metformin group withdrew from the study. The main reasons for withdrawal were AEs and failure to follow-up (**Figure 1**).

The baseline characteristics of the patients are presented in **Table 1**. At baseline, the only significant between-group difference was observed for VFA (greater in the PEG-Loxe group, *P* < 0.001).

**Table 1:** Baseline characteristics of patients.

	PEG-Loxe(n=104)	Metformin(n=52)	*P* value
Age, y	40.3 (10.0)	42.2 (9.5)	0.25
Women, N (%)	37 (35.6)	20 (38.5)	0.72
Duration, y	2.1 (1.7)	2.1 (1.5)	0.92
Body weight, kg	87.6 (13.9)	87.9 (13.7)	0.90
BMI, kg/m^2^	30.0 (3.6)	30.1 (3.5)	0.92
WC, cm	102.1 (14.6)	104.6 (16.9)	0.34
VFA, cm^2^ [median (IQR)]	127.0 (114.0–146.5)	113.5 (100.0–134.8)	<0.001
HbA1c, %	8.79 (0.83)	8.68 (0.95)	0.46
FPG, mmol/L	8.56 (0.87)	8.46 (0.96)	0.52
C-peptide, nmol/L [median (IQR)]	2.40 (1.65–2.90)	2.00 (1.40–2.88)	0.43
HOMA2-%B	122.1 (40.8)	119.3 (44.9)	0.70
HOMA2-%S [median (IQR)]	16.3 (13.5–23.8)	18.8 (12.9–27.7)	0.47
TC, mmol/L	5.05 (0.71)	5.36 (1.13)	0.09
TG, mmol/L	1.88 (0.53)	1.79 (0.52)	0.32
LDL-C, mmol/L	3.50 (0.66)	3.64 (0.80)	0.26
HDL-C, mmol/L [median (IQR)]	1.40 (1.20–1.65)	1.55 (1.11–1.80)	0.90
SBP, mmHg [median (IQR)]	132.0 (136.0–138.8)	134.0 (128.0–136.0)	0.59
DBP, mmHg [median (IQR)]	78.0 (70.0–81.5)	78.0 (70.3–80.0)	0.53
AST, U/L	44.7 (19.3)	39.6 (16.9)	0.12
ALT, U/L	48.8 (17.1)	45.2 (16.6)	0.23
Pulse rate, bpm [median (IQR)]	76.0 (69.0–82.0)	76.5 (70.0–83.0)	0.51
Previously treated with:			
lifestyle interventions, %	83 (79.8)	43 (82.7)	0.67
OAD therapy, %	21 (20.2)	9 (17.3)	0.67

BMI, body mass index; WC, waist circumference; VFA, visceral fat area; HbA1c, glycated hemoglobin; FPG, fasting plasma glucose; HOMA2-%B, updated homeostatic model assessment for beta cell function; HOMA2-%S, updated homeostatic model assessment for insulin sensitivity; TC, total cholesterol; TG, triglycerides; LDL-C, low-density lipoprotein cholesterol; HDL-C, high-density lipoprotein cholesterol; SBP, systolic blood pressure; DBP, diastolic blood pressure; AST, aspartate aminotransferase; ALT, alanine aminotransferase; bpm, beats per minute; OAD, oral antidiabetic drug; IQR, interquartile range. Data are expressed as mean (SD), unless otherwise indicated.

### 3.1 Body weight

**Figure 2:**
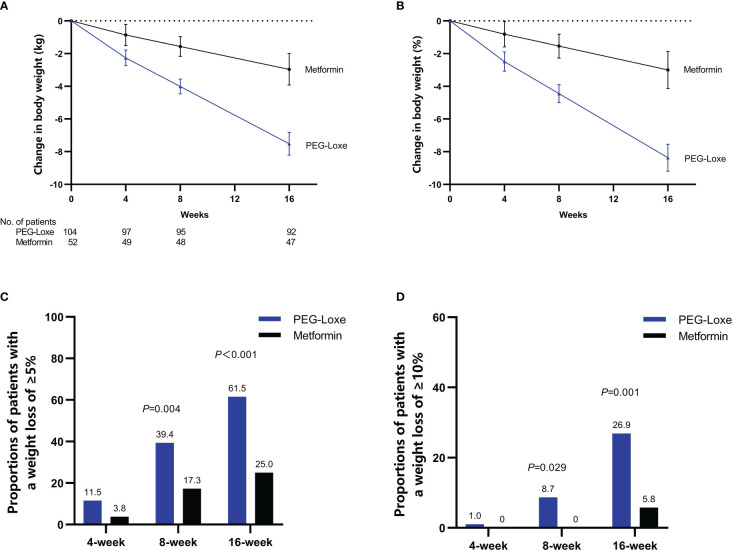
Efficacy endpoints during the 16-week treatment period: time course of absolute **(A)** and relative **(B)** changes in body weight; proportion of patients achieving ≥5% **(C)** or ≥10% **(D)** weight loss. Error bars indicate 95% CIs.

PEG-Loxe treatment resulted in significant weight loss compared to metformin treatment during the trial period. After 16 weeks, the primary endpoints (least-square mean (LSM) weight loss) were 7.52 kg (8.37%) and 2.96 kg (3.00%) for the PEG-Loxe and metformin groups, respectively, with a between-group mean difference of 4.55 kg (95% CI: 3.43, 5.67; *P* < 0.001) (**Figures 2A, B**; **Table 2**). Sensitivity analyses showed similar findings (**Supplementary Table S1**).

**Table 2:** Primary and secondary endpoints at week 16.

	PEG-Loxe Group (n=104) Mean (95% CI)	Metformin Group (n=52) Mean (95% CI)	Between-Group Difference (95% CI)	*P* value
Body weight, kg	-7.52 (-8.21, -6.82)	-2.96 (-3.92, -2.00)	-4.55 (-5.67, -3.43)	<0.001
Body weight, %	-8.37 (-9.20, -7.55)	-3.00 (-4.13, -1.86)	-5.38 (-6.68, -4.07)	<0.001
BMI, kg/m^2^	-2.55 (-2.74, -2.37)	-0.92 (-1.17, -0.67)	-1.63 (-1.92, -1.35)	<0.001
WC, cm	-12.26 (-12.98, -11.55)	-5.67 (-6.60, -4.75)	-6.59 (-7.58, -5.60)	<0.001
VFA, cm^2^	-26.02 (-27.60, -24.44)	-12.39 (-14.45, -10.32)	-13.63 (-15.85, -11.42)	<0.001
HbA1c, %	-1.22 (-1.38, -1.06)	-1.17 (-1.39, -0.96)	-0.05 (-0.28, 0.19)	0.69
FPG, mmol/L	-1.46 (-1.57, -1.34)	-1.49 (-1.65, -1.34)	0.03 (-0.14, 0.21)	0.70
HOMA2-B, %	53.23 (47.38, 59.07)	40.36 (32.63, 48.08)	12.87 (4.40, 21.35)	0.003
HOMA2-S, %	0.41 (-0.52, 1.34)	4.88 (3.64, 6.12)	-4.47 (-5.84, -3.10)	<0.001
TC, mmol/L	-0.46 (-0.52, -0.39)	-0.19 (-0.27, -0.11)	-0.27 (-0.36, -0.18)	<0.001
TG, mmol/L	-0.39 (-0.44, -0.35)	-0.19 (-0.26, -0.13)	-0.20 (-0.27, -0.13)	<0.001
LDL-C, mmol/L	-0.43 (-0.49, -0.36)	-0.19 (-0.27, -0.11)	-0.23 (-0.33, -0.14)	<0.001
HDL-C, mmol/L	-0.16 (-0.21, -0.11)	-0.12 (-0.19, -0.06)	-0.04 (-0.10, 0.03)	0.25
SBP, mmHg	-3.18 (-3.78, -2.58)	-0.28 (-1.06, 0.51)	-2.90 (-3.77, -2.03)	<0.001
DBP, mmHg	-1.34 (-1.63, -1.06)	-0.19 (-0.57, 0.19)	-1.15 (-1.59, -0.72)	<0.001

BMI, body mass index; WC, waist circumference; VFA, visceral fat area; HbA1c, glycated hemoglobin; FPG, fasting plasma glucose; HOMA2-%B, updated homeostatic model assessment for b-cell function; HOMA2-%S, updated homeostatic model assessment for insulin sensitivity; TC, total cholesterol; TG, triglycerides; LDL-C, low-density lipoprotein cholesterol; HDL-C, high-density lipoprotein cholesterol; SBP, systolic blood pressure; DBP, diastolic blood pressure.

After 16 weeks, the proportions of patients with a weight loss of ≥5% were 61.5% and 25.0% in the PEG-Loxe and metformin groups (*P* < 0.001), respectively, and those of patients with a weight loss of ≥10% were 26.9% and 5.8% in the PEG-Loxe and metformin groups (*P* =0.001), respectively (**Figures 2C, D**).

### 3.2 BMI, WC, and VFA

The LSM (95% CI) change in BMI from baseline to week 16 was greater in the PEG-Loxe group [-2.55 (-2.74, -2.37) kg/m^2^] than in the metformin group [-0.92 (-1.17, -0.67) kg/m^2^] (*P* < 0.001). The LSM (95% CI) change in WC from baseline to week 16 was greater in the PEG-Loxe treated patients [-12.26 (-12.58, -11.55) cm] than in metformin-treated patients [-5.67 (-6.60, -4.75) cm] (*P* < 0.001). After 16 weeks, VFA reduction was significantly higher in the PEG-Loxe group [-26.02 (-27.60, -24.44) cm^2^] than in the metformin group [-12.39 (-14.45, -10.32) cm^2^] (*P* < 0.001) (**Table 2**).

### 3.3 Glucose control

The LSM changes in HbA1c after week 16 were similar between the two groups (PEG-Loxe, -1.22% [95% CI: -1.38 to -1.06]; metformin, -1.17% [95% CI: -1.39, -0.96]; *P* =0.69). No significant differences were observed in the mean FPG reduction relative to the baseline between the groups at week 16 (PEG-Loxe, -1.46 mmol/L [95% CI: -1.57, -1.34]; metformin, -1.49 mmol/L [95% CI: -1.65, -1.34]; *P* =0.70). HOMA2-B levels increased in both groups; the change in the PEG-Loxe group was greater than that in the metformin group (*P* =0.003). In addition, HOMA2-S increased in both groups, with greater changes observed in the metformin group than the PEG-Loxe group (*P* < 0.001) (**Table 2**).

### 3.4 Lipid profile and blood pressure

TC, TG, and LDL-C levels were improved with PEG-Loxe treatment compared to metformin treatment (*P* < 0.001). SBP was reduced by 3.18 mmHg and and 0.28 mmHg with PEG-Loxe and metformin, respectively (*P* < 0.001). DBP was reduced by 1.34 mmHg and 0.19 mmHg with PEG-Loxe and metformin, respectively (*P* < 0.001) (**Table 2**). **Supplementary Table S2** also shows these efficacy variables at weeks 4 and 8.

### 3.5 Safety evaluation

After 16 weeks of treatment, the incidence of AEs was 46.2% (48/104) and 44.2% (23/52) in the PEG-Loxe and metformin groups, respectively (*P* =0.82). The incidence of SAEs was 2.9% (3/104) and 1.9% (1/52) in the PEG-Loxe and metformin groups, respectively. No deaths occurred during the study period. The most common AEs during the 16-week treatment were gastrointestinal disorders, with a greater incidence in the PEG-Loxe group (24.0%) than in the metformin group (17.3%) (**Table 3**). Gastrointestinal disorders were mostly mild to moderate and occurred primarily during the first four weeks of treatment. In the PEG-Loxe group, the incidence of gastrointestinal disorders was 11.5%, 5.8%, and 6.7% with 0.1 mg, 0.2 mg, and 0.3 mg treatment, respectively. Acute pancreatitis was not reported during this trial. In addition, the incidence of hypoglycemic events was 2.9% (3/104) and 3.8% (2/52) in the PEG-Loxe and metformin groups, respectively. No level 3 hypoglycemia was reported. Eight patients, five (4.8%) in the PEG-Loxe group and three (5.8%) in the metformin group, discontinued treatment because of AEs.

**Table 3:** Summary of safety.

	PEG-Loxe, No. (%) (n=104)	Metformin, No. (%) (n=52)	*P* value
Any AE	48 (46.2)	23 (44.2)	0.82
Any SAE			>0.99
Death	0 (0)	0 (0)	>0.99
Other	3 (2.9)	1 (1.9)	>0.99
AE by severity			
Severe	5 (4.8)	3 (5.8)	>0.99
Moderate	9 (8.7)	3 (5.8)	0.75
Mild	22 (21.2)	10 (19.2)	0.78
Discontinuation because of AEs	5 (4.8)	3 (5.8)	>0.99
AEs reported in ≥5% of patients by SOC/PT			
Gastrointestinal disorders	25 (24.0)	9 (17.3)	0.34
Nausea	13 (12.5)	4 (7.7)	0.43
Vomiting	6 (5.8)	1 (1.9)	0.43
Diarrhea	3 (2.9)	2 (3.8)	>0.99
Metabolism and nutritional disorders	8 (7.7)	4 (7.7)	>0.99
Decreased appetite	6 (5.8)	2 (3.8)	0.72
Infections and infestations	7 (6.7)	5 (9.6)	0.52
Upper respiratory tract infection	5 (4.8)	4 (7.7)	0.48
Hypoglycemia	3 (2.9)	2 (3.8)	>0.99
Level 1	3 (2.9)	1 (1.9)	>0.99
Level 2	0 (0)	1 (1.9)	0.33
Level 3	0 (0)	0 (0)	>0.99

AE, adverse event; SAE, serious adverse event; SOC, system organ class; PT, preferred term.

At week 16, the change in ALT was -6.88 U/L (95% CI: -7.54, -6.22) with PEG-Loxe and -2.33 U/L (95% CI: -3.17, -1.48) with metformin (*P* < 0.001). The changes in AST were -6.01 U/L (95% CI: -6.59, -5.43) with PEG-Loxe and -1.74 U/L (95% CI: -2.51, -0.97) with metformin (*P* < 0.001). Slight increases in pulse rate were observed in both groups: 2.07 bpm in the PEG-Loxe group and 0.43 bpm in the metformin group (*P* < 0.001) (**Table 2**).

## 4 Discussion

This is the first trial specifically designed to examine the efficacy of PEG-Loxe for weight management and the first trial to investigate PEG-Loxe at a higher dose of 0.3 mg with a fixed-dose-escalation regimen in patients with T2DM. A few studies have reported that a weight-maintenance phase occurred after approximately 12–16 weeks of GLP-1RA treatment (23–26); therefore, the treatment period was designed for 16 weeks to observe the short-term weight loss effect of PEG-Loxe. In the present trial, compared with metformin treatment, the addition of PEG-Loxe with lifestyle interventions or OAD therapy resulted in significantly greater weight loss at week 16. This was accompanied by significant improvements in several cardiovascular risk factors in overweight or obese patients with T2DM.

In previous phase 1–3 clinical trials, which enrolled patients with BMIs of 25–27 kg/m^2^, 0.1 mg, 0.2 mg, and 0.3 mg PEG-Loxe demonstrated a weak effect on weight loss (0.35–1.90 kg) after 8–24 weeks of treatment (15–17). In contrast, in the present trial, 0.3 mg PEG-Loxe achieved a significant weight reduction of 7.52 kg. Inconsistencies in these findings may be related to baseline characteristics of the patients. Patients with T2DM and obesity who initially had a higher BMI showed greater weight loss when they underwent GLP-1RA treatment (27). In comparison with previous trials, a higher baseline BMI (30 kg/m^2^) in the present trial might have resulted in better weight loss.

Whether directly or indirectly, obesity contributes to the development of several cardiovascular risk factors and comorbidities, including dyslipidemia, hypertension, and cardiovascular disease (28, 29). Weight loss in the 5%–10% range or more is associated with improvements in these conditions (30). The Chinese Diabetes Society recommends short-term goals for weight management involving 5%–10% of weight loss in 3–6 months in overweight and obese patients with T2DM (31). In the present trial, the proportion of patients achieving clinically meaningful levels of weight loss (≥5% or 10%) was significantly higher with PEG-Loxe than with metformin after 16 weeks of treatment.

The glycemic control effects of 0.1 mg and 0.2 mg PEG-Loxe have been well established in patients with T2DM, as shown by HbA1c reduction ranging from 1.02–1.36% following 12 or 24 weeks of treatment (14–16). Furthermore, in a phase 1 trial, the change in HbA1c was 0.9% after 8 weeks of treatment with 0.3 mg PEG-Loxe (17). Using the same dose in the present trial, similar improvements in glycemic control were observed, with HbA1c reductions of 1.22% after 16 weeks of treatment. Moreover, in the present trial, PEG-Loxe and metformin displayed almost equal potency for glycemic control. The dose of metformin administered in this trial was 1500 mg/day, which is the conventional and most widely used dose in China. This dose conferred an HbA1c reduction of 1.17%, similar to a previous report wherein metformin reduced the HbA1c level by approximately 1.0–1.5% in patients with T2DM (31).

The safety profile of PEG-Loxe in the present trial was consistent with that in previous trials (15, 16). No new safety concerns have been identified. The main AEs associated with PEG-Loxe were gastrointestinal disorders (24.0%), which were predominantly mild to moderate and mainly occurred in the first 4 weeks (15, 16). To date, reports of a dose-escalation regimen for PEG-Loxe are not yet available. This present trial used a fixed-dose-escalation regimen to reduce possible gastrointestinal disorders. Consequently, 0.3 mg PEG-Loxe did not cause a significant increase in gastrointestinal disorders, and the incidences were comparable to those observed in previous trials using 0.1–0.2 mg PEG-Loxe (10.3–25.0%) (15, 16). In addition, GLP-1RAs do not increase the risk of hypoglycemia because of their glucose-dependent antidiabetic effects (32, 33). The incidence of hypoglycemic events in the present trial was comparatively low, and no level 3 hypoglycemia was reported. Additionally, GLP-1RAs are known to increase resting heart rate (34); in this trial, 0.3 mg PEG-Loxe increased the mean pulse rate by 2.07 bpm, which appears to be a class side effect of GLP-1RAs.

This study had several limitations. The open-label design of the present trial may have increased the risk of bias. Furthermore, the clinical trial duration was relatively short, and the full potential of 0.3 mg PEG-Loxe efficacy on weight loss may have been missed. Therefore, additional trials are warranted to assess whether these effects are maintained with long-term 0.3 mg PEG-Loxe treatment.

In conclusion, once-weekly subcutaneous PEG-Loxe administration resulted in significantly greater weight loss at 16 weeks, when compared with metformin administration, in overweight or obese patients with T2DM, with lifestyle interventions or OAD therapy. The effects of PEG-Loxe on body weight may provide a treatment option for overweight or obese patients with T2DM.

## Data availability statement

The raw data supporting the conclusions of this article will be made available by the authors, without undue reservation.

## Ethics statement

The studies involving human participants were reviewed and approved by the Ethics Committee of Shandong Provincial Hospital. The patients/participants provided their written informed consent to participate in this study.

## Author contributions

YD and XZ designed the trial. HC, QC, and XZ conducted the trial. HC and QC collected the data. XZ interpreted the data. YZ performed data analysis. HC, QC, and YD drafted the manuscript. XZ reviewed the manuscript. All authors approved the final version of the manuscript.
