# Supplementary material for: Short-term effect of polyethylene glycol loxenatide on weight loss in overweight or obese patients with type 2 diabetes: An open-label, parallel-arm, randomized, metformin-controlled trial
Source: Front Endocrinol (Lausanne). 2023 Jan 26;14:1106868. doi: 10.3389/fendo.2023.1106868 (PMC9909427; doi:10.3389/fendo.2023.1106868)
Supplement: Supplementary file 1 [file DataSheet_1.docx]

Supplementary Material

Figure S1


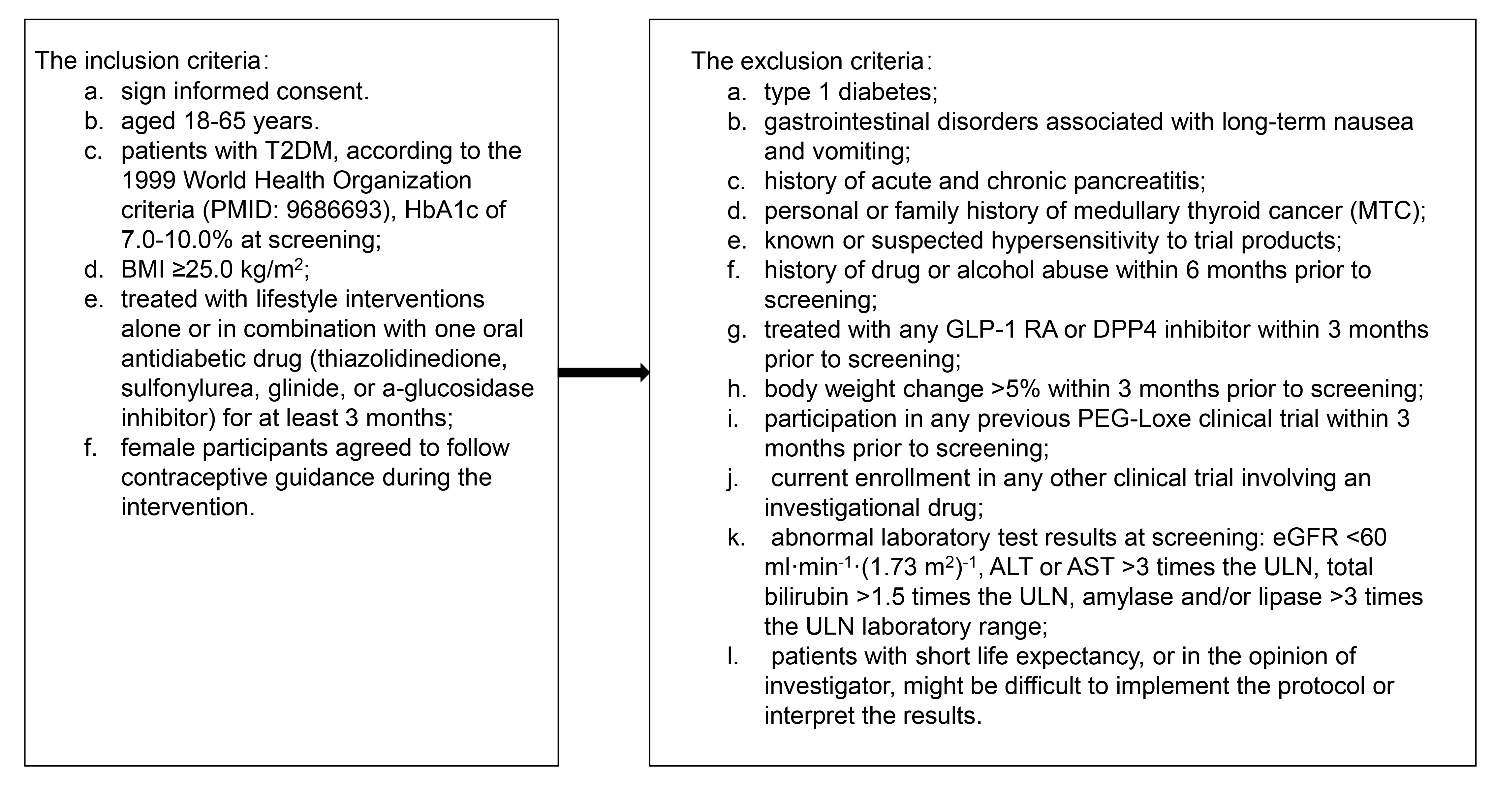


Figure S1 | The inclusion and exclusion criteria.

Figure S2


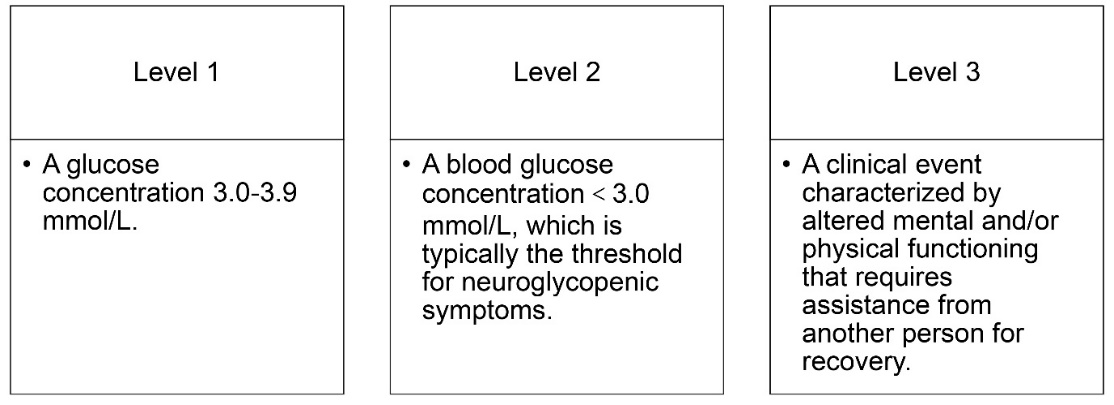


Figure S2 | Classification of hypoglycemia

Table S1 | Sensitivity analyses of the primary endpoint at week 16 (per-protocol set).

|  | PEG-Loxe Group | |  |  | Metformin Group | |  |  |
| --- | --- | --- | --- | --- | --- | --- | --- | --- |
|  | No. of  patients | Mean  (95% CI) |  |  | No. of  patients | Mean  (95% CI) | Between-Group Difference  (95% CI) | *P* value |
| Change in body weight, kg | 92 | -7.50 (-8.02, -6.98) |  |  | 47 | -2.77 (-3.47, -2.08) | -4.73 (-5.52, -3.94) | ＜0.001 |
| Change in body weight, % | 92 | -8.36 (-8.97, -7.74) |  |  | 47 | -2.80 (-3.61, -1.98) | -5.56 (-6.46, -4.66) | ＜0.001 |

Table S2 | Summary of the efficacy variables at week 4 and week 8.

|  | PEG-Loxe Group  (n=104)  Mean (95% CI) |  | Metformin Group  (n=52)  Mean (95% CI) | Between-Group Difference  (95% CI) | *P* value |
| --- | --- | --- | --- | --- | --- |
| Change in body weight, kg |  |  |  |  |  |
| Week 4 | -2.26 (-2.74, -1.78) |  | -0.86 (-1.51, -0.21) | -1.40 (-2.11, -0.70) | ＜0.001 |
| Week 8 | -4.01 (-4.46, -3.56) |  | -1.56 (-2.16, -0.96) | -2.45 (-3.10, -1.81) | ＜0.001 |
| Change in body weight, % |  |  |  |  |  |
| Week 4 | -2.49 (-3.07, -1.90) |  | -0.81 (-1.59, -0.03) | -1.68 (-2.51, -0.85) | ＜0.001 |
| Week 8 | -4.45 (-5.00, -3.90) |  | -1.54 (-2.27, -0.81) | -2.91 (-3.67, -2.15) | ＜0.001 |
| Change in BMI, kg/m^2^ |  |  |  |  |  |
| Week 4 | -0.77 (-0.89, -0.64) |  | -0.23 (-0.40, -0.06) | -0.54 (-0.71, -0.36) | ＜0.001 |
| Week 8 | -1.36 (-1.49, -1.24) |  | -0.46 (-0.62, -0.30) | -0.90 (-1.07, -0.74) | ＜0.001 |
| Change in WC, cm |  |  |  |  |  |
| Week 4 | -3.54 (-4.08, -2.99) |  | -0.99 (-1.67, -0.31) | -2.54 (-3.16, -1.92) | ＜0.001 |
| Week 8 | -6.44 (-6.98, -5.91) |  | -2.55 (-3.21, -1.90) | -3.89 (-4.47, -3.31) | ＜0.001 |
| Change in VFA, cm^2^ |  |  |  |  |  |
| Week 4 | -8.62 (-9.86, -7.38) |  | -3.86 (-5.39, -2.32) | -4.76 (-6.20, -3.32) | ＜0.001 |
| Week 8 | -14.42 (-15.62, -13.21) |  | -6.70 (-8.19, -5.21) | -7.72 (-9.07, -6.32) | ＜0.001 |
| Change in HbA1c, % |  |  |  |  |  |
| Week 4 | -0.36 (-0.49, -0.23) |  | -0.39 (-0.56, -0.22) | 0.04 (-0.14, 0.21) | 0.69 |
| Week 8 | -0.64 (-0.77, -0.52) |  | -0.65 (-0.81, -0.49) | 0.01 (-0.14, 0.16) | 0.92 |
| Change in FPG, mmol/L |  |  |  |  |  |
| Week 4 | -0.54 (-0.63, -0.46) |  | -0.62 (-0.73, -0.51) | 0.08 (-0.03, 0.19) | 0.15 |
| Week 8 | -0.85 (-0.93, -0.77) |  | -0.91 (-1.02, -0.81) | 0.07 (-0.04, 0.17) | 0.21 |
| Change in HOMA2-%B |  |  |  |  |  |
| Week 4 | 17.67 (13.27, 22.07) |  | 17.09 (11.48, 22.70) | 0.58 (-4.82, 5.99) | 0.83 |
| Week 8 | 29.53 (25.28, 33.77) |  | 24.85 (19.51, 30.19) | 4.68 (-0.30, 9.66) | 0.07 |
| Change in HOMA2-%S, % |  |  |  |  |  |
| Week 4 | 0.48 (-0.21, 1.17) |  | 1.51 (0.62, 2.40) | -1.03 (-1.91, -0.16) | 0.02 |
| Week 8 | 0.45 (-0.21, 1.12) |  | 2.63 (1.79, 3.48) | -2.18 (-2.99, -1.37) | ＜0.001 |
| Change in TC, mmol/L |  |  |  |  |  |
| Week 4 | -0.16 (-0.21, -0.11) |  | -0.04 (-0.10, 0.02) | -0.12 (-0.18, -0.06) | ＜0.001 |
| Week 8 | -0.26 (-0.31, -0.21) |  | -0.09 (-0.15, -0.03) | -0.17 (-0.22, -0.12) | ＜0.001 |
| Change in TG, mmol/L |  |  |  |  |  |
| Week 4 | -0.12 (-0.15, -0.09) |  | -0.04 (-0.08, 0.01) | -0.08 (-0.13, -0.04) | ＜0.001 |
| Week 8 | -0.21 (-0.24, -0.18) |  | -0.09 (-0.13, -0.05) | -0.12 (-0.27, -0.13) | ＜0.001 |
| Change in LDL-C, mmol/L |  |  |  |  |  |
| Week 4 | -0.11 (-0.15, -0.06) |  | -0.05 (-0.11, 0.01) | -0.06 (-0.11, 0.00) | 0.06 |
| Week 8 | -0.21 (-0.26, -0.17) |  | -0.10 (-0.15, -0.04) | -0.12 (-0.17, -0.06) | ＜0.001 |
| Change in HDL-C, mmol/L |  |  |  |  |  |
| Week 4 | -0.05 (-0.09, -0.02) |  | -0.04 (-0.09, 0.00) | -0.01 (-0.05, 0.03) | 0.69 |
| Week 8 | -0.09 (-0.12, -0.05) |  | -0.07 (-0.11, -0.03) | -0.02 (-0.06, 0.02) | 0.35 |
| Change in SBP, mmHg |  |  |  |  |  |
| Week 4 | -0.99 (-1.43, -0.56) |  | 0.23 (-0.33, 0.79) | -1.22 (-1.76, -0.68) | ＜0.001 |
| Week 8 | -1.72 (-2.15, -1.30) |  | 0.06 (-0.47, 0.60) | -1.78 (-2.29, -1.28) | ＜0.001 |
| Change in DBP, mmHg |  |  |  |  |  |
| Week 4 | -0.36 (-0.58, -0.16) |  | 0.04 (-0.23, 0.30) | -0.39 (-0.66, -0.12) | 0.004 |
| Week 8 | -0.69 (-0.88, -0.49) |  | -0.04 (-0.29, 0.21) | -0.65 (-0.90, -0.40) | ＜0.001 |

Abbreviations: BMI, body mass index; WC, waist circumference; VFA, visceral fat area; HbA1c, glycated hemoglobin; FPG, fasting plasma glucose; HOMA2-%B, homeostasis model assessment index 2 for b-cell function; HOMA2-%S, homeostasis model assessment index 2 for insulin sensitivity; TC, total cholesterol; TG, triglycerides; LDL-C, low-density lipoprotein cholesterol; HDL-C, high-density lipoprotein cholesterol; SBP, systolic blood pressure; DBP, diastolic blood pressure.
